# Efficient Synchronous Extraction of Nickel, Copper, and Cobalt from Low–Nickel Matte by Sulfation Roasting‒Water Leaching Process

**DOI:** 10.1038/s41598-020-66894-x

**Published:** 2020-06-18

**Authors:** Qiangchao Sun, Hongwei Cheng, Xiaoyong Mei, Yanbo Liu, Guangshi Li, Qian Xu, Xionggang Lu

**Affiliations:** State Key Laboratory of Advanced Special Steel & Shanghai Key Laboratory of Advanced Ferrometallurgy & School of Materials Science and Engineering, Shanghai University, Shanghai, 200444 People’s Republic of China

**Keywords:** Physical chemistry, Process chemistry

## Abstract

Considering that the low recovery efficiency and the massive loss of valuable metals by the traditional pyrometallurgical process smelting low‒nickel matte. Therefore, this paper focuses on studying the optimal process parameters and the mechanism of sulphation roasting followed by water leaching achieving efficient synchronous extraction of nickel (Ni), copper (Cu), and cobalt (Co) from low‒nickel matte with sodium sulfate as the sulfating addictive. Under optimal conditions, the recovery efficiency of Ni, Cu, and Co metals can achieve 95%, 99%, and 94%, respectively, whereas the recovery efficiency of Fe metal is less than 1%. The results revealed that the mechanism of the sulfating roasting pretreatment could form a liquidus eutectic compound sulfates [Na_2_Me(SO_4_)_2_] (Me = Ni, Cu, Co) at the solid–solid interface, which plays a significant role in promoting the leaching efficiency of valuable metals. Not only enhance the reaction kinetics of sulfation, but improve the utilization efficiency of SO_2_/SO_3_. Thus, the sulfation roasting‒water leaching process developing an efficient and eco-friendly pathway to simultaneous extraction of Ni, Cu, and Co valuable metals from low grade sulfide ores.

## Introduction

Nickel metal as an important strategic resource, it has been widely applied in batteries, catalysts, and other fields thanks to the extraordinary physicochemical properties. Currently, the sulfide ore are the predominant raw materials for refining extract nickel metal^1–3^. Unfortunately, the grades of sulfide ores are falling as the higher grade reserves are exploited first and are progressively depleted due to the increasing demand for applications. Thus, improving the extraction efficiency and comprehensive recycling valuable metals from nickel resources is a sustainable strategy to meet the demand for nickel^4,5^.

Low‒nickel matte as the main intermediate product of nickel sulfide ore in traditional pyrometallurgical smelting, during this process, the valuable metals Ni, Cu, and Co have been enriched^6,7^. In the traditional smelt route, the low‒nickel matte are further converter blowing to reduce the iron content and other impurities forming the high nickel‒copper matte. However, through this procedures, almost 70 wt % Co and most part Ni, Cu are transferred to the converter slag, leading to the massive loss of valuable metals. Furthermore, the environmental issues of the emissions of oxysulfide gases in the pyrometallurgical process are inevitably. Consequently, developing an efficient and environmentally friendly method to synchronous extraction of nickel, copper, and cobalt from low‒nickel matte is a highlighted issue^8–10^.

With the low‒nickel matte as raw materials to extract the valuable metals by hydrometallurgical method, which not only can simplify the process, also avoid the loss of Ni, Co in the traditional blowing process^11^. In general, the hydrometallurgical process of sulfide ore including the heap leaching^12^, acid leaching^13^, and ammonia leaching^14,15^. However, there are many drawbacks in the treatment of sulfide ore by single pyrometallurgy or hydrometallurgy process (such as high energy consumption, complicated processing, and the poor selectivity of valuable metals), specially, in the case of atmospheric acid leaching. In order to remove these obstacles, the pre–roasting‒leaching combination process as an effective strategy have been applied extensively owing to the energy consumption saved and processes simplified. During the pre–roasting process, commonly the used additives including ammonium chloride^16–18^, ammonium sulfate^3,19,20^, sodium sulfate^21,22^ and other roasting additives^23^ to enhance the roasting effect. Benefitting from the low-cost, noncorrosive nature, and high metal leaching selectivity of sodium sulfate, it has been considered as the promising roasting additive to enhance the extraction efficiency and selectivity in the later leaching process.

However, since the miscellaneous chemical reactions sparking during the calcination process of complex polymetallic minerals, thus, the mechanism of sodium sulfate as an additive in the roasting process has not been clearly defined. Generally, there are two kinds of controversy^21,22,24^: one holds that the sodium sulfate reacts with the sulfide ore to form the corresponding metal sulfate, accompanied by the formation of sodium sulfide, then the sodium sulfide react with O_2_ regenerates sodium sulfate, thus the sulfation mechanism of sodium sulfate is similar to the catalyst function. The corresponding reaction formulas are as follows:

1 $$MeS+N{a}_{2}S{O}_{4}=MeS{O}_{4}+N{a}_{2}S$$

2 $$N{a}_{2}S+2{O}_{2}=N{a}_{2}S{O}_{4}$$

The another view hold that sodium sulfate can form eutectic compound sulfate (Na_2_Me(SO_4_)_2_) react with the preformed metal sulfate (such as nickel sulfate, copper sulfate, and cobalt sulfate), and the formation of the eutectic compound sulfate can not only improve the stability of the metal sulfate, but also serve as the storage for SO_2_ (SO_3_). The corresponding processes can be summarized by the following equations:

3 $$N{a}_{2}S{O}_{4}+MeS{O}_{4}=MeS{O}_{4}\cdot N{a}_{2}S{O}_{4}$$

4 $$S{{O}_{4}}^{2-}+S{O}_{3}={S}_{2}{{O}_{7}}^{2-}$$

5 $$MeO+{S}_{2}{{O}_{7}}^{2-}=M{e}^{2+}+2S{{O}_{4}}^{2-}$$

Till now, the behavior of sodium pyrosulfate has yet to be discussed, because the direct proof has not been given.

In this work, we investigated the sulfation roasting of low‒nickel matte at the condition of sodium sulfate added, then transferring nickel, copper, and cobalt to the aqueous solution by water leaching. A series of crucial parameters of the roasting temperature, amount of oxygen, sodium sulfate addition amount, and heating rate are optimized to achieve efficiently separation of valuable metals. Moreover, the phase transition during roasting was analyzed by TG‒DSC and XRD. The microstructure of the roasting product and the leaching residue and the migration of each element during roasting were characterized by SEM‒EDS. The mechanism of sodium sulfate revealing that sodium sulfate plays a prominent role in the sulfation roasting of complicated polymetallic sulfide ores.

## Experiments

### Sulfation roasting–water leaching process

All the samples used in this experiment are provided by Jinchuan Group Ltd., in Gansu Province, China. The minerals are crushed and screened to obtain –200 mesh powder samples (<74 μm), then dried at 100 °C for 48 h.

**Figure 1:**
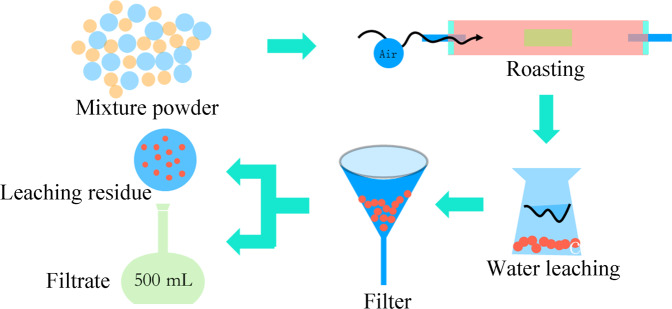
Schematic diagram of sulfation roasting‒water leaching.

1.0 g of low–nickel matte and 0.1 g of sodium sulfate were mixed in an agate mortar, then transferred to the corundum crucible for sulfation roasting in a tube furnace. After roasting, the roasting products were transferred to the 250 mL conical flask, 150 mL of deionized water was added, and then the conical flask was placed in a constant temperature (90 °C) water bath for 1 h with strong agitation. It was filtered after the completion of the water bath, then the filter residue was rinsed with deionized water for several time, placed in a petri dish for drying in an oven. The filtrate was transferred to the 500 mL volumetric flask. The schematic diagram of this sulfation roasting**–**water leaching was illustrated in Fig. 1. After leaching, the leaching efficiency of different metal elements are calculated, respectively, according to calculation formula as follows:

6 $${\eta }_{x}=\frac{{C}_{x}V}{{m}_{0}{\omega }_{x}}\times 100 \% $$

where C_x_ is the concentration of metal in the leachate, mg/L, V is the volume of the volumetric flask, L, m_0_ is the total mass of the mineral in the experiment, g, and ω_x_ is the percentage of the corresponding metal element in the mineral, %. For minimizing the error, the experiment was repeated three times in each group and the average value was employed.

### Characterization of phase transition and element migration

The crystalline structure characterization of the roasting products and leached residues were investigated by the Bruker New D8 Advance X–ray diffractometer couple with the Cu Kα radiation (λ = 0.154 nm). The results were collected with the scanning rate at a speed of 4°/min and the scanning range of 2θ from 10° to 80° (40 kV, 40 mA). The concentration of the metal element in the leaching solution was determined by the inductively coupled plasma optical emission spectroscopy (ICP–OES**/**PERKINE 7300DV, Perkin Elmer) to obtain the accurate extraction efficiency of different metal. The scanning electron microscopy (SEM/HITACHI SU–6700) couple with the energy dispersive spectroscopy (EDS) was carried out to study the microscopic surface morphology features and structure of samples. The thermogravimetry-differential scanning calorimetry (TG–DSC/NETZSCH STA 449 F3) was performed to determine the approximate range of the actual reaction and the endothermic situation of the low‒nickel matte and the mixture with additives. The conditions of this experiment are that the sample was placed in an Ar atmosphere (40 ml/min), and the temperature was raised from 50 °C to 1000 °C with a rate of 5 °C/min.

## Results and Discussion

### Components identification of low‒nickel matte

**Figure 2:**
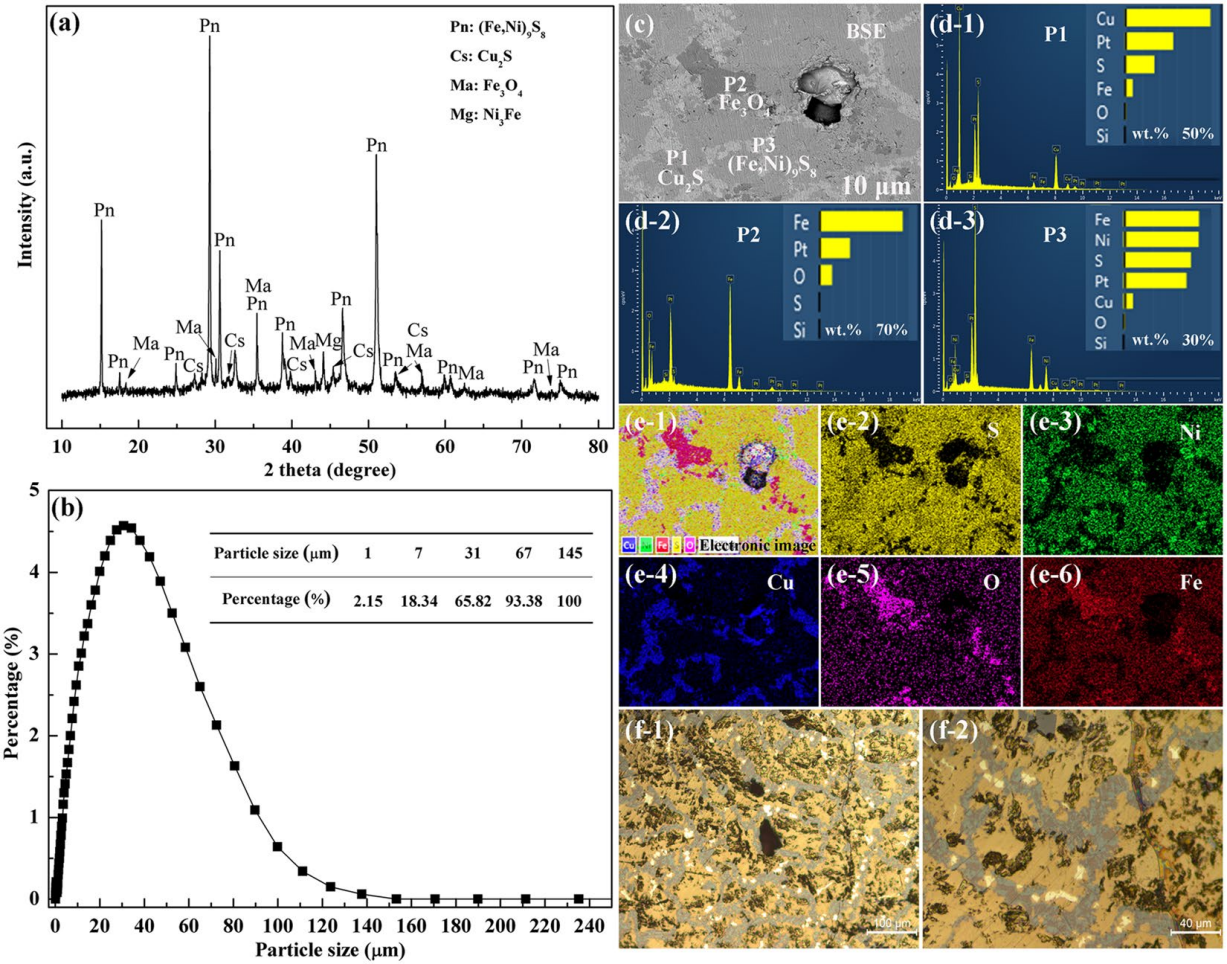
(**a**) XRD pattern of the low–nickel matte, (**b**) particle size distribution of low–nickel matte powders, (**c**‒**e**) element distribution of low–nickel matte, (**f**) metallographic images of low–nickel matte.

The elemental compositions of the sample is measured by ICP–OES and XRF, and the results are given in Table 1. Obviously, the main metallic elements of sample are the (Fe, Ni, Cu) was determined by X–ray fluorescence (XRF) and inductively coupled plasma (ICP) measurements. Figure 2 shows the phase composition and microstructure of the low‒nickel matte. The dominant component are the pentlandite ((Fe,Ni)_9_S_8_), chalcocite (Cu_2_S), and a small amount of magnetite (Fe_3_O_4_) and iron–nickel alloy (Ni_3_Fe). The particle size distribution of low‒nickel matte powder after sieving is presented in Fig. 2b. Comprehensive analyze the energy spectrum (Fig. 2(c–e)) and the metallographic (Fig. 2(f)) found that the chalcocite main distributed in the grain boundary in a band form, while the matrix phase is the pentlandite and the small amount of bright white spots are the iron oxide black. Those results are fit well with that of XRD analyses.

**Table 1:** Elemental composition in low–nickel matte (wt %).

	S	O	Ni	Fe	Cu	Co	Zn	Si
XRF	17.21	2.16	27.89	27.11	24.98	0.49	0.05	0.04
ICP–OES	—	—	25.21	30.27	19.10	0.46	—	—

Based on the above fundamental measurements of low–nickel matte. The key parameters of sulfation roasting are systematically studied in the following section. The schematic diagram of sulfation roasting‒water leaching is shown in Fig. 1 and the details of roasting conditions of per group are presented in Table 2.

**Table 2:** The detail roasting conditions of each group.

	Fig. 3(a)	Fig. 3(b)	Fig. 3(c)	Fig. 3(d)	Fig. 3(e)	Fig. 3(f)
Temperature (°C)	400–800	400–800	600	600	600	600
Na_2_SO_4_ addition (wt %)	0	10	0–100	10	10	10
Oxygen content (%)	21	21	21	0–21	21	21
Heating rate (°C/min)	2	2	2	2	2–6	2
Holding time (min)	120	120	120	120	120	0–180

### Effect of temperature

**Figure 3:**
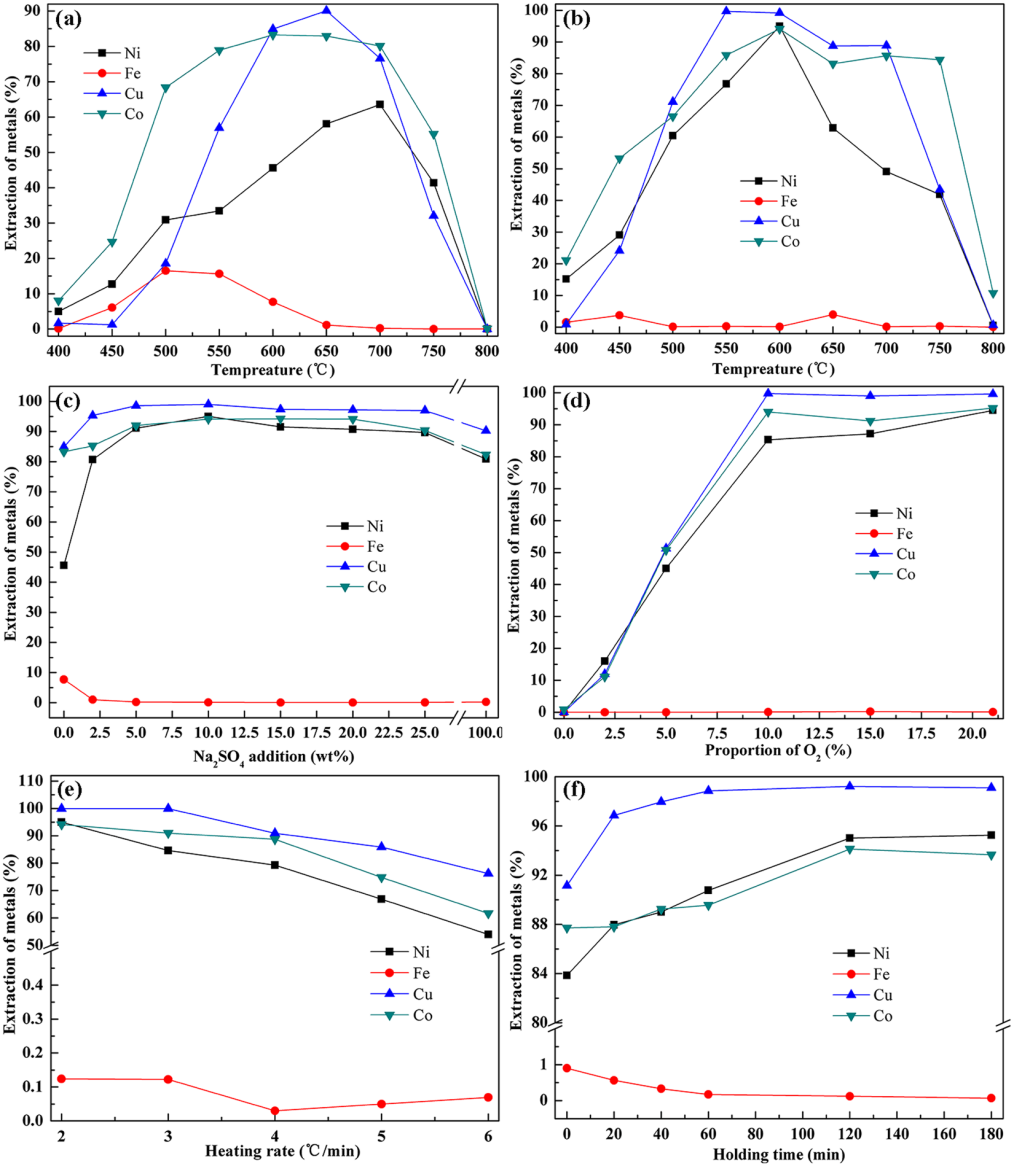
Metal extraction efficiency with different roasting parameters, (**a**) the effect of temperature on the extraction of metals, (**b**) the effect of temperature on the extraction of metals, (**c**) the effect of dosage of sodium sulfate on the extraction of metals, (**d**) the effect of oxygen proportion on the extraction of metals, (**e**) the effect of heating rate on the extraction of metals, (**f**) the effect of holding time on the extraction of metals. (Notes: (**a**) without sodium sulfate added, (**b**‒**f**) with sodium sulfate added).

Firstly, the effect of roasting temperature on the extractions efficiency of nickel, copper, cobalt, and iron was studied, the results were shown in Fig. 3a,b. the Fig. 3a exhibits the leaching results without sodium sulfate added. With the temperature increasing, the extraction efficiency of different metals showing a variation trend of increasing first and then decreasing, which results are consistent well with the previous reports^19^. The leaching yields of Co, Cu, and Ni first reached a maximum are 86%, 90%, and 63% at 600 °C, 650 °C, and 700 °C, respectively. However, comparing to Cu and Co, the extraction of Ni requires higher temperature whereas the leaching yield is relatively low as well, which show the poor Ni leaching selectivity under direct roasting–water leaching conditions. Furthermore, the leaching yield of Fe reached maximum at 500 °C, and then decreases with increase of temperature, the leaching yield of that is less than 0.25% at 700 °C, which is attributed to the poor thermal stability of Ferric sulfate^25^.

Figure 3b shows the variation of metal leaching yield with temperature with sodium sulfate added in roasting process. Obviously, the leaching yield of Cu is less than 1% at 400 °C, while the leaching yields of Ni and Co are 15% and 21%, respectively. This result is the same as the discipline of low–nickel matte without the addition of sodium sulfate. Since the Ni and Co metals have higher affinity with oxygen, and they are superior to Cu metal preferential oxidation. However, the recovery percent of Ni and Co are higher than those without sodium sulfate at same conditions, which revealing that the addition of sodium sulfate has already enhanced the roasting effect at 400 °C. The leaching yield of Ni, Cu, and Co are rising in the range of 400–600 °C, it reached a maximum at 600 °C with 95%, 99%, and 94%, respectively. Particularly, the maximum leaching yield of Ni increased by 32% at lower temperature. After 600 °C, the leaching yield of Ni, Cu, and Co are all show the downward trend, and the decreasing trend of leaching yield of nickel is particularly sensitive to temperature. Moreover, the leaching yield of iron reaches a maximum at 450 °C and 650 °C, respectively. The former may owing to the formation of transitional soluble sulfate by sulfating of ferrous oxide at relatively low temperature, while the latter is due to the rapid decomposition of sulfate of nickel, copper and cobalt at high temperature resulting in a large amount of SO_2_, which leading the reaction (7) proceed to the opposite direction. Therefore, the addition of Na_2_SO_4_ addictive can significantly enhance the low–nickel matte sulfate roasting and the optimum roasting temperature is 600 °C.

7 $$2F{e}_{2}{(S{O}_{4})}_{3}=2F{e}_{2}{O}_{3}+6S{O}_{2}+3{O}_{2}$$

### Effect of dosage of sodium sulfate

Based on the aforementioned results, the addition of sodium sulphate is crucial factor for improving selectivity and efficiency of the metal in leaching process. Thus, we studied the influence of dosage of sodium sulphate on extraction of Co, Cu, and Ni from low–nickel matte. The results are shown in Fig. 3c. the leaching yields of Ni, Cu, and Co reached the maximum when the dosage of sodium sulfate is approximately 10 wt %, and its interesting that the higher extraction efficiency of Cu (about 98%) than Co and Ni, considering the different chalcography of Cu, Co, and Ni in the low-nickel matte, we ascribed to the result of Cu_5_FeS_4_ species were easier oxidation dissolution than that of (Fe, Ni)_9_S_8_ species^11^. The improvement of extraction efficiency may attribute to the decomposition of sulfides have been promoted with sodium sulfate added. However, when the dosage exceed 10 wt %, its influences on the extraction efficiency of Ni, Cu, and Co can be ignored. Results indicate that the addition of sodium sulfate in an appropriate dosage can enhance the extraction efficiency of Ni, Cu, and Co. Therefore, the optimal dosage is chosen as 10 wt %.

### Effect of oxygen contents

The previous literature have provided that the predominance area diagram for Me–S–O (Me = Ni, Cu, Fe, Co) system at constant temperature^22^. Generally, the initial step in the sulfation process is oxidizing reaction. The predominance area diagram reveals that the reaction product is determined by the gas phase composition. Therefore, it is necessary to discuss the effect of oxygen content on roasting. Figure 3d described the effect of oxygen content on metals’ leaching yields. Known from Fig. 3d, the extraction efficiency of Ni, Cu, and Co increase significantly with the increase of oxygen content as the oxygen proportion is below 10%. And they reached maximum values (98%, 94%, and 85%, respectively), when the proportion of O_2_ is 10%. But the extraction efficiency of Fe is hardly change with the increase of oxygen content. When the oxygen content exceeds 10%, the extraction efficiency of Cu and Co does not change substantially, while the extraction efficiency of Ni showed a continuous increase trend, indicating that Cu is preferred sulfation to the Ni. Results demonstrated that O_2_ is beneficial to the sulfation process only in a certain range concentration, if its proportion exceeded the range, the oxidation dissolution reaction of low–nickel matte will be weaken. Nevertheless, taking costs into consideration we choose the 21% O_2_ experimental atmosphere (in the air) was used in the following experiments.

### Effect of heating rate

The kinetics of the sulfation roasting process lie the heart part of extracting valuable metals from low–nickel matte. Thereby, its essential to perform exploration on the role of the heating rate in sulfation roasting. As seen from Fig. 3e, the leaching efficiency of Ni, Cu, and Co decreases with the increase of the heating rate, while the leaching efficiency of Fe is close to zero. The reason for the declining efficiency of Ni, Cu, and Co with the increase of the heating rate maybe the local overheating occur caused by highly exothermic oxidation reaction, resulting in a large amount of spinel phase ferrite (Ni, Cu, and Co) Fe_2_O_4_ formation^26^. Thus, the suitable heating rate is essential to maintain the uniform temperature profile guarantee the efficient sulfation roasting process.

### Effect of soaking time

The experimental results of the leaching efficiency of each metal with different soaking time are demonstrated in Fig. 3f. Before the 120 min of soaking time, the leaching efficiency of Ni, Cu, and Co gradually increase with the holding time prolonged and then reach a plateau. Specially, as the holding time extended from 0 min to 20 min, the leaching efficiency of Ni and Co increase substantially, which may ascribe to the enhanced sulfation of NiS, NiO, and some spinel phase by–products during the soaking time^27^. As for iron, with the prolongation of the holding time, the leaching efficiency of Fe is reverse to that of Ni, Cu, and Co. In addition, when the holding time exceeds 120 min, the leaching efficiency of Fe is also reach a plateau (nearly 0%). Therefore, the optimum of holding time should be maintained 120 min. The detail behavior and mechanism of Na_2_SO_4_ in heating and soaking processes will be discussed in following parts.

### Phase transition in roasting process

**Figure 4:**
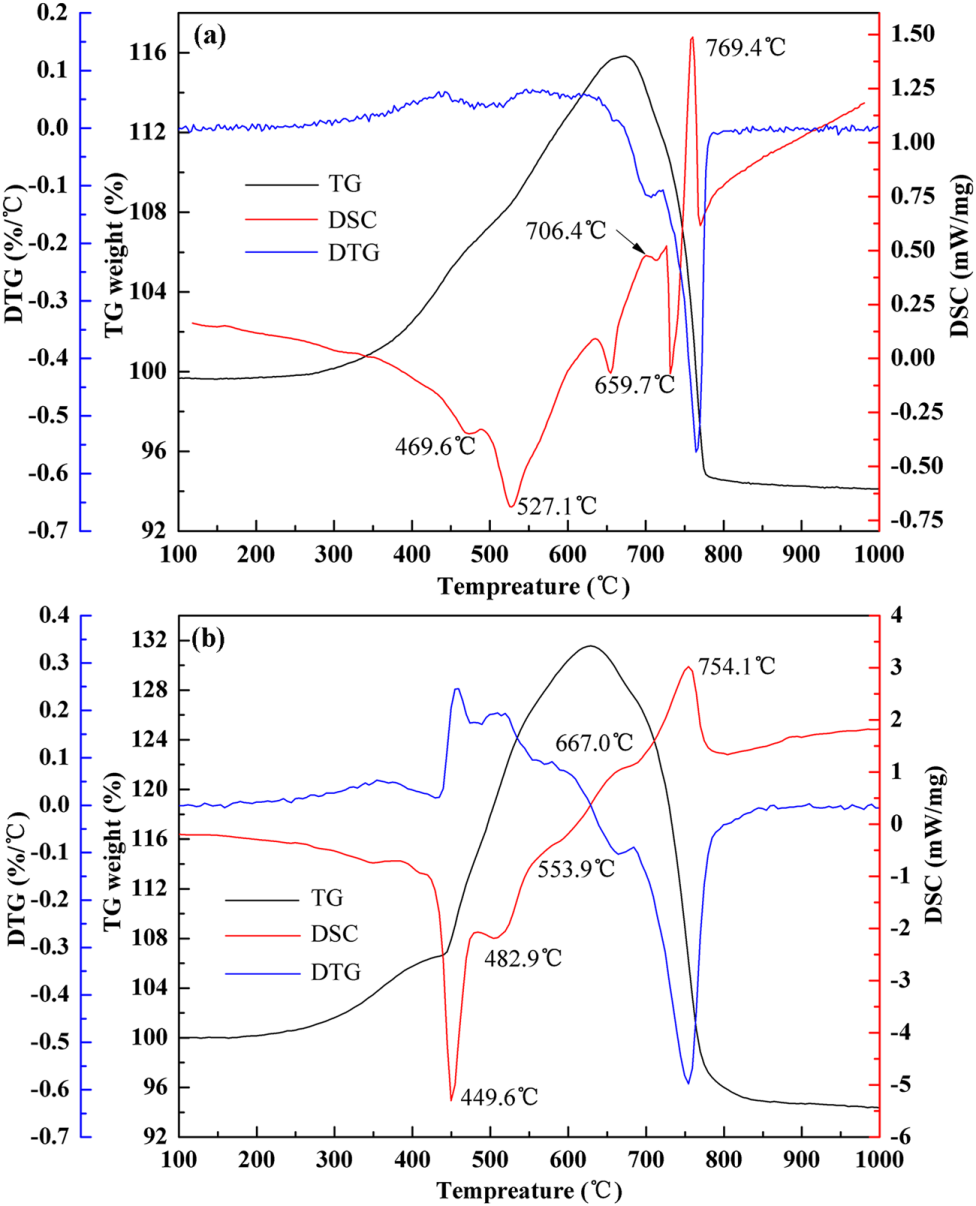
Thermal analysis curve of low–nickel matte, (**a**) without sodium sulfate, (**b**) with sodium sulfate.

Based on the optimal process parameters confirmed, it found that sodium sulfate could facilitate the sulfation reaction dynamics of low–nickel matte and resulting in the markedly promoted extraction efficiency and selectivity of Ni, Co, and Cu. Thus, in order to have an insight into the phase transition during sulfation roasting, The TG–DSC tests were conducted to detect the mixture and then the roasting products were analyzed by XRD. The TG–DSC curves of low–nickel matte mix with or without sodium sulfate samples were carried out with a heating rate (5 °C/min) from 50 to 1000 °C. The results are shown in Fig. 4.

### TG–DSC characterization

Figure 4a shows the thermal analysis curve of low‒nickel matte without sodium sulfate. There are three exothermic peaks in the process of mass increase, which are related to the oxidation of metal sulfides. The two endothermic peaks during the weight loss process, which are ascribed to the decomposition of sulfates. Its noteworthy that when the temperature exceed 400 °C, the process of gradual oxidative desulfurization of metal sulfides to form metal sulfates contributing the TG curve of sample increased sharply. However, when the temperature higher than 700 °C, the TG curve of sample decline remarkably. Those results are consistent with the change law of different metal’s leaching yield. Figure 4b illustrated the thermal analysis curve of low–nickel matte with sodium sulfate added. Comparing to Fig. 4a, the corresponding endothermic and exothermic temperatures are all moving to the low temperature direction. In addition, the net mass increase is 31.56% with sodium sulfate as addictive, which is twice as high as that of 15.83% without sodium sulfate added, indicating that the addition of Na_2_SO_4_ might increase the stability of sulfates.

### XRD characterization

**Figure 5:**
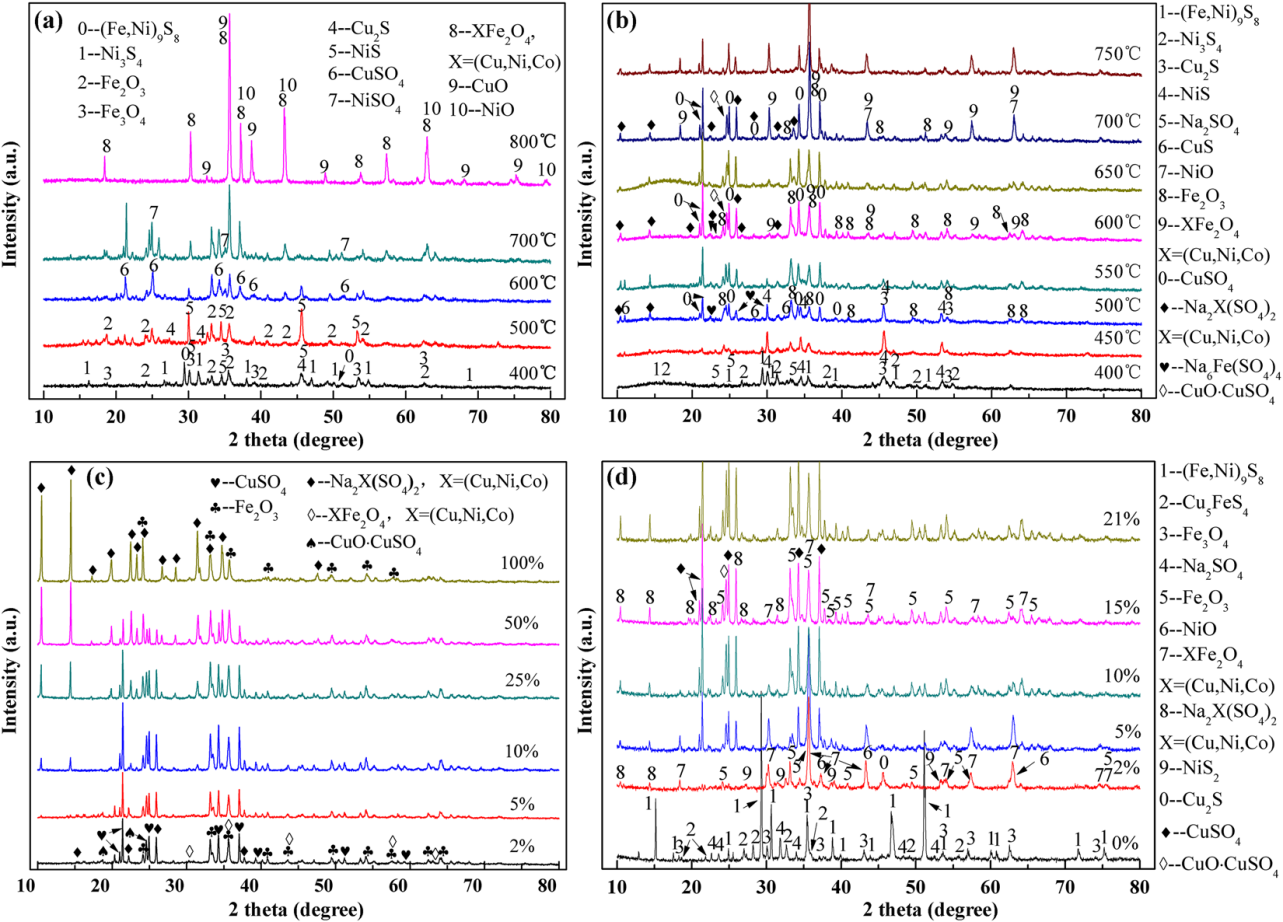
XRD spectrum of the roasting products, (**a**) roasting at different temperatures without sodium sulfate added, (**b**) roasting at different temperatures with sodium sulfate added, (**c**) roasting at 600 °C with different dosage of sodium sulfate, (**d**) roasting at 600 °C with different oxygen proportion (with sodium sulfate added).

In combination with the XRD crystal phase analysis of products obtained from different roasting conditions, which is better to understand the potential chemical reactions and its level comprehensively during the different roasting processes. Figure 5 represents the XRD patterns of different roasting products.

Figure 5a displays the roasting products of low‒nickel matte at various temperatures. At relative low temperature (400 °C), obviously, the pentlandite and chalcocite are mainly phase composition. With the increase of temperature, the pentlandite gradually oxidize to the monometallic sulfides. When it reaches 600‒700 °C temperature range, NiSO_4_ and CuSO_4_ are the dominant compositions of the roasting products, corresponding to the high extraction efficiency of copper and nickel metals in water leaching process (Fig. 3a). However, with the temperature heats up to 800 °C, the sulfates (NiSO_4_, CuSO_4_) gradually decompose to form the corresponding metal oxides, meanwhile, the major spinel phase MeFe_2_O_4_ (Me = Ni, Cu, and Co) emerged, which are more difficult to leaching from roasting products. Accounting for the decrease of metal leaching yields in the temperature range (Fig. 3a). As for iron, the appearance of ferric oxides diffraction peak at 400 °C, indicates that the iron is preferentially oxidized at initial oxidation stage. Fortunately, the ferric oxides may act as a catalyst for the subsequent sulfation processes, the results have been reported by previous literatures^28,29^. Its worth noting that no diffraction peaks of ferric sulfate have been detected at any temperature, which may be related to its inferior thermal stability. Resulting the decomposition of ferric sulfate and generating SO_2_ gases in the early roasting stage that is beneficial for the sulfation of nickel and copper^30^.

Figure 5b exhibits the low–nickel matte roasting products’ XRD patterns with 10 wt % sodium sulfate as additive. Similarly, the oxidative decomposition processes of pentlandite and chalcocite are coincident to that without sodium sulfate added. At lower temperature (400 °C) the diffraction peaks of sodium sulfate are remained. Based on the above TG‒DSC tests, the amounts of soluble sulfates are gradually increase with the temperature increasing. Its worth pointing out that, no nickel sulfate phases are detected among all samples. However, the new phases of composite sulfates (Na_2_Ni(SO_4_)_2_) are formed, which may because of the generated metal sulfates reacted with Na_2_SO_4_ immediately at higher temperature that means higher kinetics for reactions. In conjunction with Fig. 5c, its believed that the increase of compound sulfate phases is the reason for higher leaching yield of nickel metal in Fig. 3b.

Figure 5d illustrates the XRD patterns of roasting products under different proportion of O_2_ conditions. Apparently, the pentlandites are unchanged even at 600 °C with absence of O_2_, while the chalcocite converted into bornite phase. With the increase of O_2_ contents, the products contain major spinel phase MeFe_2_O_4_ (like NiFe_2_O_4_), metal oxides (NiO and Fe_2_O_3_), and metal sulfide (NiS_2_), which further confirm that poor reaction kinetics of sulfation because of the insufficient O_2_ concentration. A suitable O_2_ concentration feed is essential to the formation of composite sulfates (Na_2_Ni(SO_4_)_2_) with Na_2_SO_4_ added.

### SEM and EDS characterization

**Figure 6:**
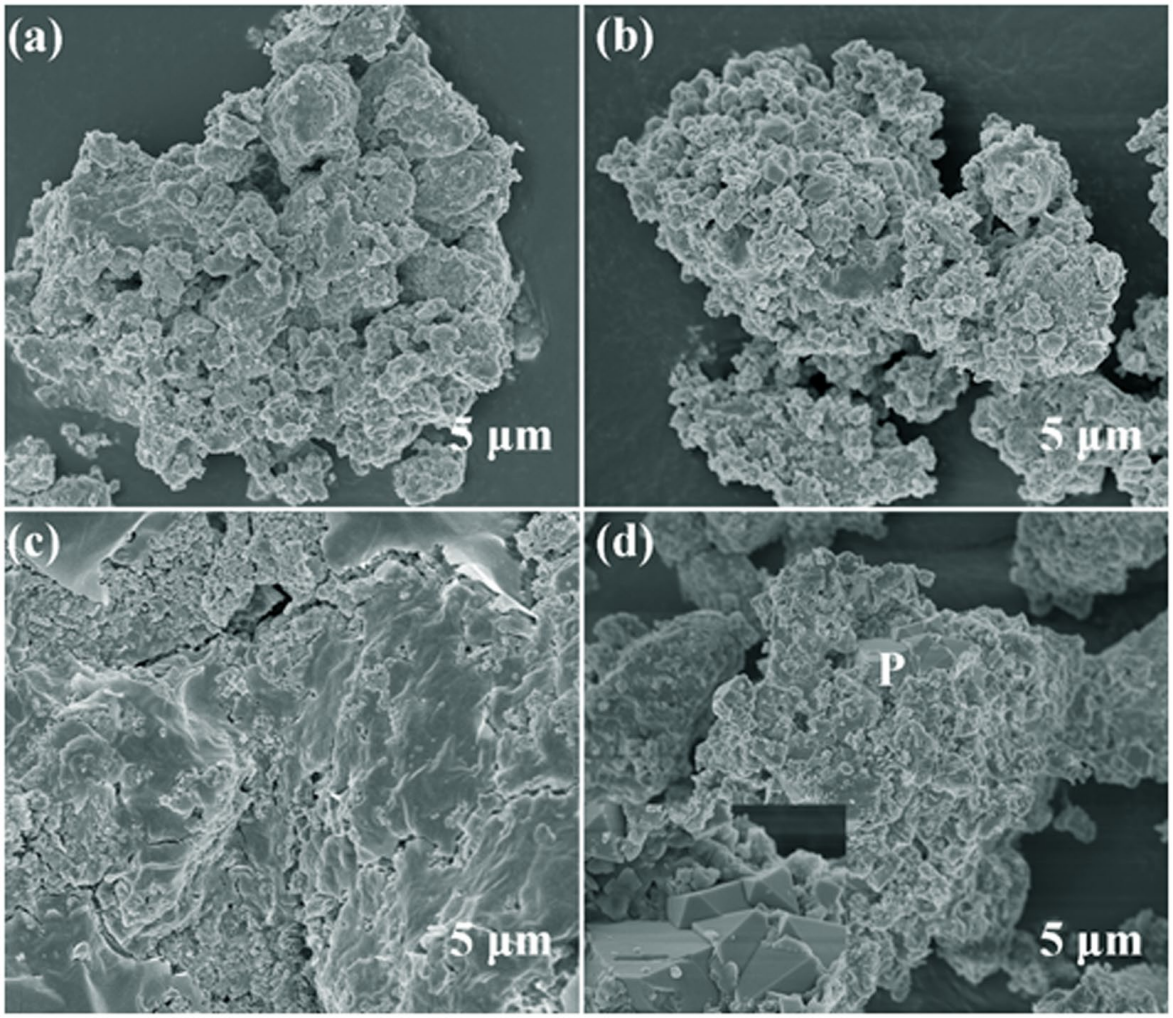
SEM image of roasting products at different temperatures, 10% sodium sulfate added, (**a**) 400 °C, (**b**) 500 °C, (**c**) 600 °C, (**d**) 700 °C.

Figure 6 demonstrates the microscopic morphology of low–nickel matte after roasting at different temperatures with 10% sodium sulfate is added. At 400 °C, the mineral phases have changed basically, there are many porous particles are formed on the surface. As the roasting temperature reached 500 °C, with the progression of the oxidation reaction and sulfation roasting, a trace of the sintered phase formed between porous particles, which is generally considered to be the formation of a composite mineral sulfates during the roasting process. Moreover, this type binary sulfides and/or solid solution composite mineral sulfates usually exists with a liquid form in the reaction conditions^31^. Thus, resulting in the agglomeration of particles form sintered phase. In addition, the proportion of this low melting complex mixture of binary sulfates and sulfate solid solution phases increase with the roasting temperature raising, and that has been demonstrated that the liquid phases composite mineral sulfates formed could maintain the high stability during the sulfation roasting process and prevent non-ferrous metals sulfate from decomposition^32,33^. However, with the temperature reached 700 °C, the complex sulfates decompose and the spinel phase formed consequently. The inset is the zoom-in image of the P region in Fig. 6d, this is a typical spinel phase structure, which is consistent with the XRD results.

**Figure 7:**
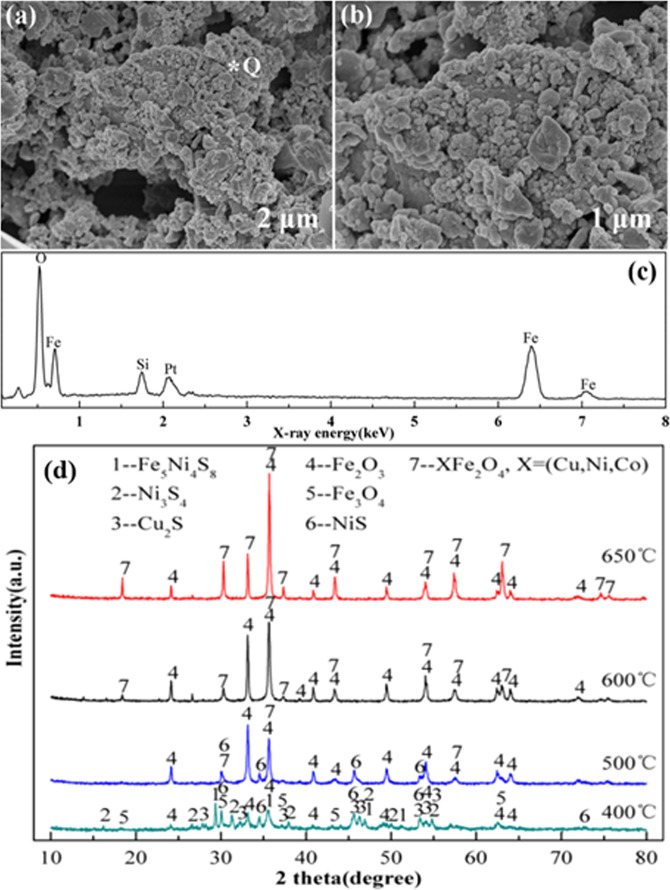
SEM + EDS and XRD patterns of leaching residue, **(b**) zoom‒in image of (**a**,**c**) is the energy spectrum of point Q of (**a**,**d**) shows the XRD patterns of leaching residue with different temperature.

Figure 7a,b show the surface morphology of the leaching residue after sulfation roasting at 600 °C and the morphology is consisted of agglomerated microspheres clusters. Combining with the energy spectrum (Fig. 7c) and the XRD pattern (Fig. 7d) show that the leaching residue are mainly iron oxide species, owing to the most of iron are preferentially oxidized to form iron oxides layer(s) at the initial roasting stage, leading to the separation of Ni/Cu and Fe through complicated reactions and ion diffusion. Indicating that the high leaching selectivity of non‒ferrous metals from sulfation roasting products except for the iron metal under optimum conditions.

Taking the above results and analysis into consideration, the sulfation roasting process of low–nickel matte can be divided into three stages. In the first stage, the pentlandites are oxidized and decomposed into monometallic sulfide, then converting to the ferric oxide and ferric sulfate (the reverse reaction of reaction (7)). Related literature have reported that during the oxidation of pentlandites, the surface iron is firstly oxidized to form iron–deficient pentlandites^30^, then form the simple oxides and sulfides. The possible reactions are as follows:

8 $${(Fe,Ni)}_{9}{S}_{8}+{O}_{2}\to F{e}_{2}{O}_{3}+{(Fe,Ni)}_{9-x}{S}_{8-y}+S{O}_{2}$$

9 $${(Fe,Ni)}_{9-x}{S}_{8-y}+{O}_{2}\to F{e}_{2}{O}_{3}+N{i}_{3}{S}_{4}+NiS+S{O}_{2}$$

**Figure 8:**
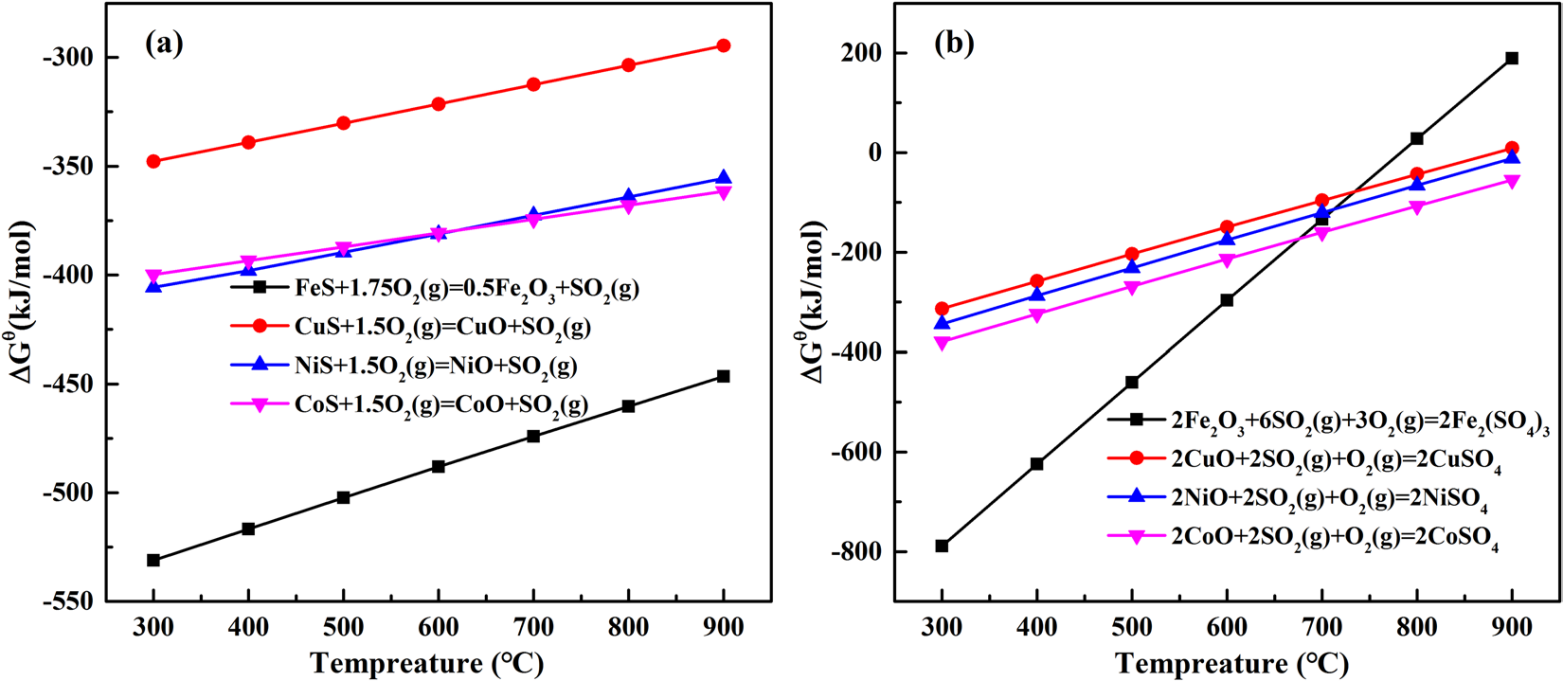
Reaction thermodynamic calculation (**a**) the oxidation of Fe, Ni, Cu, and Co sulfides, (**b**) the sulfation of Fe, Ni, Cu, and Co oxides.

In the second stage, sulphation of nickel and copper sulfides and the decomposition of ferric sulphate (reaction (7)) occurs, the possible reactions are as follows, and the corresponding thermodynamic analysis are presented in Fig. 8. Thermodynamic calculations indicate that the oxidation and sulfation of Fe, Ni, Cu and other sulfides are spontaneous reactions with extremely negative Gibbs free energy (ΔG). Obviously, these reactions can be completed quickly within the appropriate range of O_2_ and SO_2_ partial pressure. The iron sulfides oxidation and iron oxides sulfation are the first to proceed, besides, the oxidation and sulfation of Ni are prior to the Cu at 600 °C, which is consistent with the experimental results.

10 $$N{i}_{3}{S}_{4}+{O}_{2}=3NiS+S{O}_{2}$$

11 $$NiS+2{O}_{2}=NiS{O}_{4}$$

12 $$2NiS+3{O}_{2}=2NiO+2S{O}_{2}$$

13 $$2NiO+{O}_{2}+2S{O}_{2}=2NiS{O}_{4}$$

14 $$2C{u}_{2}S+5{O}_{2}=2CuO+2CuS{O}_{4}$$

15 $$2CuO+{O}_{2}+2S{O}_{2}=2CuS{O}_{4}$$

With addition of sodium sulfate, the reaction (3) occurs. In the third stage, including the decomposition of nickel and copper sulphate and the formation of spinel phases (MeFe_2_O_4_), and the possible reactions are as follows:

16 $$2CuS{O}_{4}=CuO\cdot CuS{O}_{4}+S{O}_{3}$$

17 $$CuS{O}_{4}=CuO+S{O}_{3}$$

18 $$CuO+F{e}_{2}{O}_{3}=CuF{e}_{2}{O}_{4}$$

19 $$NiS{O}_{4}=NiO+S{O}_{3}$$

20 $$NiO+F{e}_{2}{O}_{3}=NiF{e}_{2}{O}_{4}$$

### Mechanism of sodium sulfate in roasting process

**Figure 9:**
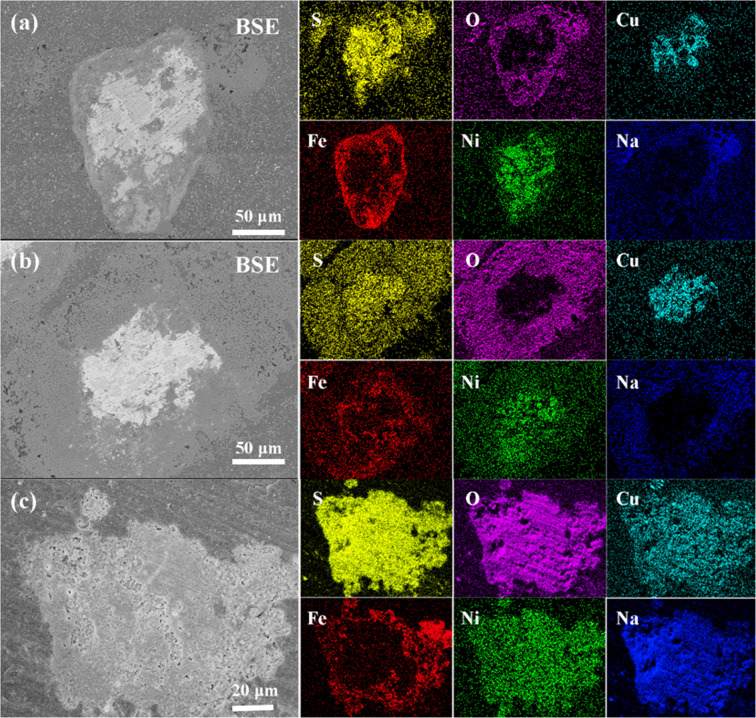
BSE image and EDS mapping of roasting products with different holding time (**a**) 20 min, (**b**) 120 min, and (**c**) 180 min.

According to the course of sulfation roasting reaction, the role and behavior of sodium sulfate in the sulfation roasting process were further investigated. Thus, the SEM and EDS measurements of roasting products’ cross section are conducted. Figure 9a–c show the results of roasting products with sodium sulfate added under different holing time (600 °C). When the holding time kept 20 min, the particles of samples consist of three distinctly layers. The outmost layer is coated with the addictive sodium sulfate, and the middle layer is the Fe_2_O_3_, because of iron preferential oxidation. The inner zone is an intermediate Cu/Ni sulfide. As the holding time increases to 120 min, with the progress of reaction (9) and (10), which would lead to the shrinkage of the Cu/Ni sulfide cores that provided more space for the formation of Cu/Ni sulfide and/or the composite sulfates (Na_2_Ni/Cu(SO_4_)_2_) and the outer layer is coated with composite mineral sulfate. Those results are coincident well to the previous XRD dates that the composite mineral sulfate phase have been detected. However, when the holding time was further increased to 180 min, the Cu/Ni sulfides were completely oxidized and sulfated, the outer composite of thin Fe_2_O_3_ layer.

Furthermore, basing on the phase diagrams of the binary sulfates NiSO_4_·Na_2_SO_4_ and CoSO_4_·Na_2_SO_4_ systems, the formation of the liquid phases compound sulfate phase Na_2_Me(SO_4_)_2_ (reaction 21, 22, and 23), which are the process of increasing the entropy (ΔS) value of the chemical reaction, so the reaction can be achieved under experimental conditions^22,34,35^. The liquid phases compound sulfates not only increased the reaction rate of copper and nickel sulfate, but formed local liquid phase in the periphery due to its low melting characteristic, which acts as a buffer layer for the gas (SO_2_, O_2_) in and out, resulting in the formation of ferrite was inhibited. Meanwhile, it has been discussed in previous literature that nickel sulfites formed during roasting exhibits a dense core–shell structure that hinders the further reaction of nickel sulfides^21^.

21 $$N{a}_{2}S{O}_{4}+MeS{O}_{4}=N{a}_{2}Me{(S{O}_{4})}_{2}(l)$$

22 $$N{a}_{2}S{O}_{4}+NiS+2{O}_{2}=N{a}_{2}Ni{(S{O}_{4})}_{2}(l)$$

23 $$N{a}_{2}S{O}_{4}+NiO+S{O}_{2}+1/2{O}_{2}=N{a}_{2}Ni{(S{O}_{4})}_{2}(l)$$

This explains a phenomenon mentioned above: nickel sulfides takes precedence over copper sulfides in thermodynamics during sulfation reaction, but the nickel sulfides are inferior to the copper sulfides at higher temperature. The formation of eutectic compound sulfate (reaction 21, 22, and 23) broken the dense core–shell structure of nickel sulfate, which promoting the sulfation reaction kinetics of NiO and NiS. Thus, resulting in the higher formation kinetics of nickel sulfate that’s beneficial to the formation of the composite sulfates (Na_2_Ni/Cu(SO_4_)_2_). In addition, the fluidized layer would change the original gas‒solid, solid–solid two phase reaction become a gas–solid–liquid three phase reaction, which greatly improved the dynamics of reactions. This explained why the addition of sodium sulfate greatly improving the recovery of nickel/copper.

24 $$2S{O}_{2}+{O}_{2}=2S{O}_{3}$$

**Figure 10:**
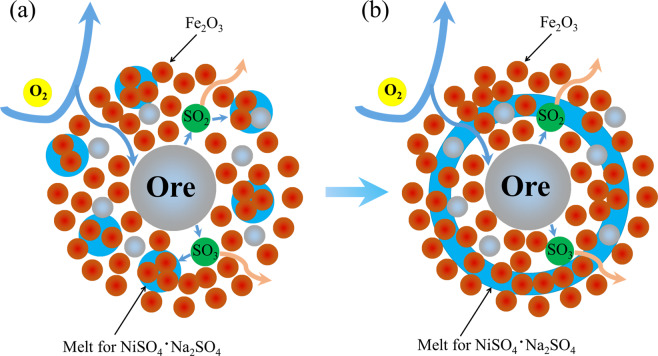
Schematic of the sulfation mechanism of low–nickel matte with the addition of sodium sulfate (**a**) a suitable dosage of sodium sulfate (forming a local liquid phase layer), (**b**) an excessive addition of sodium sulfate as a sulfation additive.

The formation of ferric oxides could accelerate with the reaction (24) proceed, thereby boosting the sulfation process of nickel and copper. Then the nickel and copper sulfates reacted with sodium sulfate in the roasting process formed eutectic compound sulfate. Under roasting conditions, the compound sulfate exists with liquid form and cover the unreacted Cu/Ni sulfides cores. Oxygen diffused through the liquid phase to reacts with the central Cu/Ni sulfides. The formed SO_2_ (SO_3_) are also shielded by this liquid phase, thus the gas cannot escape, which increases the utilization ratio of sulfur dioxide and maximizes the degree of sulfation process. Besides, the local liquid phase buffer layer could redistribute the oxygen before it reacts with central Cu/Ni sulfides, which not only would prevent the massive formation of ferrates due to severe oxidation of Cu/Ni sulfides, but also improve the stability of Cu/Ni sulfides. The overall sulfation mechanism is schematically represented by the Fig. 10a. But it is worth noting that if there is too much the liquid phase eutectic compound sulfate formed, the liquid phase layer with certain thickness could hinder the gases and ion diffusion, as shown in Fig. 10b, which would produce negative effects during sulfation roasting process. That also explained the problem of a slight decrease in the metal leaching yield when the dosage of sodium sulfate reaches 100% in Fig. 3c.

In conclusion, simultaneous extraction of Ni, Cu, and Co through the sulphation roasting followed the water leaching strategy with cost–effective, high efficiency, and eco–friendly process. Several experimental parameters have been studied systematically, *i.e*., the effects of temperature, heating rate, dosage of sodium sulfate, and holding time. After water-leaching the Ni, Cu, and Co in the form of sulfate dissolve into the solution, but the Fe with the form of ferric oxides remain in the leaching residue, thereby achieving efficient selective separation of Ni, Cu, and Co from Fe. Under optimum sulfation roasting conditions (roasting temperature 600 °C, holding time 2 h, heating rate 2 °C/min, 10% sodium sulfate addition) the leaching efficiency of Ni, Cu, and Co reach as high as 95%, 99%, and 94%, respectively. The contribution of sodium sulfate have been demonstrated that sodium sulfate could react with nickel sulfate and copper sulfate formed liquid phase eutectic compound sulfate, which provided the appropriate kinetics environment for sulfation roasting, thereby maximizing the extraction efficiency of Ni, Cu, and Co from low–nickel matte.
